# The history of the semantic hacking project and the lessons it teaches for modern cognitive security

**DOI:** 10.3389/frai.2025.1616447

**Published:** 2025-09-24

**Authors:** Paul Thompson, Sean Guillory

**Affiliations:** ^1^Dartmouth College, Hanover, NH, United States; ^2^MAD Warfare, Portland, OR, United States

**Keywords:** artificial intelligence, generative AI, large language models, countermeasures, cognitive security

## Abstract

The Semantic Hacking Project ran from 2001 to 2003. It focused on how information systems (and the human decisions shaped by them) could be exploited through attacks not on code or infrastructure, but on meaning. This work is relevant to contemporary cognitive security concerns in the face of today’s information space. The work provides insight into the key question of how people come to hold the beliefs which they do. The project anticipated many of today’s challenges (disinformation campaigns, social media manipulation, AI-generated narratives) not just in technical terms, but in philosophical and linguistic terms. At the heart of its concern was a simple but powerful question: What happens when you can manipulate the inputs to a person’s belief system without the person knowing it? This question has only grown more urgent in an era of generative AI, large language models (LLMs), and algorithmically amplified influence.

## 1 Introduction: definitions as weapons

While this special issue of *Frontiers Science* will present a variety of definitions for “cognitive security,” we want to offer a conceptual framing that explains why this domain matters so deeply for national security and warfare. For us, cognitive security is not just a new subset of cybersecurity or psychological operations; it is a foundational way to understand modern conflict itself.

Warfare has never been purely about kinetic force. While what happens in the physical domain is possibly the most extreme physical and psychological experience known to man, most of the real contest happens in the cognitive domain: the space of interpretation, belief, perception, and judgment. It is in this contested arena of ideas, narratives, and sense-making (the outcome of which is decided away from psychical battlefield concerns by people not physically present in the battlefield themselves) that who really won or lost a conflict is resolved. Whether a missile hits its target, whether a nation complies with sanctions, or whether an election’s outcome is accepted by its population, all of these hinge less on what physically happens, but more on what people believe has happened and why (Perception is/as Reality).

War is itself is a communicative act where every kinetic operation sends a message, whether that message is intentional or not. A successful airstrike or special operation can achieve all of its tactical objectives and still fail strategically if the resulting narrative (from the adversaries on the battlefield, the adversaries off the battlefield, and all the civilian audiences in between) does not “hit” as planned. The moment action is taken, it becomes part of a communicative sequence, interpreted by different audiences through varying lenses. In this sense, modern war is not just about what is done, but about what it means to adversaries, allies, civilian populations, and the broader international system.

It’s understood if the reader is having difficulty understanding the what, the where, and how the cognitive domain works. It is like trying to understand the physics and biology of a Looney Tunes universe that works off of the rules of imagination itself. But trying to measure it and understand it is a must to anyone who is serious about figuring out how to win wars. People often treat fiction as fact if it aligns with their worldview; realities can be ignored; and symbols can override material truths.

And we would argue that in these fights that happen in the cognitive domain, the battles over definitions themselves may be the most important because whatever is the definition of gender, genocide, A.I., and the like that are agreed upon by the participants and audience hold the power in defining a situation and what people think should happen next. To correct the most famous quote from Dune, “He who controls the definitions, controls the universe.”

Now with that contextual layout, we get to the importance of the central concept of our paper: semantic hacking. Beginning in 2001, a project at Dartmouth College’s Institute for Security Technology Studies launched one of the earliest systematic efforts to explore how information systems could be attacked not by disrupting their hardware or code, but by manipulating the meaning of the data they carry. This effort, known as the Semantic Hacking Project, sought to understand and model the kinds of attacks that target human judgment, language, and interpretation. The project introduced the concept of “cognitive attacks” and explored a range of countermeasures aimed at defending decision-making processes from deliberate semantic distortion.

What makes this work relevant today is not just its prescience to discussions about modern cognitive security concerns in the face of today’s information space, but its insight into the key question of how people come to believe what they believe. The project anticipated many of today’s challenges (disinformation campaigns, social media manipulation, AI-generated narratives) not just in technical terms, but in philosophical and linguistic terms. At the heart of its concern was a simple but powerful question: What happens when you can manipulate the inputs to a person’s belief system without them knowing it? This question has only grown more urgent in an era of generative AI, large language models (LLMs), and algorithmically amplified influence. For example, consider current debates over whether an artificial intelligence system is “sentient.” The core of that discussion often comes down not to the system’s actual behavior, but to the *definition* of “sentient” that is adopted. Change the definition, and the outcome of the debate changes accordingly. The same applies to politically or morally loaded concepts like “life,” “gender,” “sovereignty,” or “genocide.” In each case, the battle over perception begins with a battle over language and categories. Once a term is defined in a particular way, the logical conclusions that follow often feel self-evident to those operating within that frame. But as the Semantic Hacking Project emphasized, those frames are often up for grabs, and can be subtly or overtly manipulated with the help of AI across multiple media channels at scale. Again, these were insights from almost 25 years ago.

This paper will explore the history of the Semantic Hacking Project and the intellectual trajectory it initiated, including the workshops and academic developments that followed. We will then contextualize those early insights in light of modern cognitive security challenges, particularly those raised by artificial intelligence. In doing so, we hope to demonstrate that many of the threats being discussed today are not entirely new. Rather, they are the modern instantiations of conceptual battles that have been quietly brewing for decades. We share all this because we believe that understanding this somewhat forgotten line of research could offer a useful foundation for the urgent work ahead.

## 2 The semantic hacking project: origins and intellectual contributions

The Semantic Hacking Project was one of the early efforts of Dartmouth College’s Institute for Security Technology Studies (ISTS), now known as the Institute for Security, Technology, and Society. Running from 2001 to 2003, the project was ahead of its time in its focus on how information systems (and the human decisions shaped by them) could be exploited through attacks not on code or infrastructure, but on meaning.

The term “semantic attack” had previously been introduced by Libicki (1994), who categorized computer network attacks into three types: physical (targeting hardware), syntactic (targeting system functionality, e.g., with viruses or worms), and semantic. Semantic attacks aimed not to break systems, but to manipulate decision-making whether by a human, a software agent, or an organization.

Building on this conceptual foundation, the Dartmouth team (led by George Cybenko and including Annarita Giani and co-author of this paper Paul Thompson) launched a project that would reframe these semantic threats as “cognitive attacks,” emphasizing the human interpretive processes at risk. While primarily conceptual, the project did culminate in a working prototype of one of the proposed countermeasures. More significantly, it produced one of the first structured taxonomies of cognitive threats and countermeasures in the information domain. Initial results were presented at workshops beginning in 2002, and the project’s first journal publication followed shortly thereafter (Cybenko et al., 2002a; Cybenko et al., 2002b; Thompson, 2003). A more comprehensive treatment was published in a 2004 volume of *Advances in Computers* (Cybenko et al., 2004). The team’s work remains one of the earliest and most comprehensive treatments of semantic and cognitive hacking in national security contexts.

### 2.1 Reframing the problem: from syntactic disruption to cognitive exploitation

Unlike traditional cyberattacks at the time focusing on breaking systems, cognitive attacks work by distorting how humans perceive reality and make decisions. Rather than taking down a server or corrupting data, a cognitive attack shifts the interpretation of information. The attacker does not need to control the system, they only need to control the conclusions the system’s users draw from it. In the realm of cyber, avid practitioners of “social engineering” had applied experience that led them to such insights but in terms of structured academic studies on the subject, it was The Semantic Hacking Project that categorized potential countermeasures into two types based on the structure of the information environment: those designed for single-source contexts and those designed for environments with multiple, redundant sources of information. The descriptions of the seven countermeasures are excerpted from an early Semantic Hacking project paper (Cybenko et al., 2002c).

### 2.2 Examples of single-source countermeasures

In situations where information flows from a single source (e.g., single sensor, website, or official report), the following countermeasures were proposed.

#### 2.2.1 Authentication and trust ratings

This method involved authenticating the information source and assessing its long-term reliability. Public Key Infrastructures and other certification tools could verify the identity of a source, while performance-based scoring could track its historical accuracy. Such systems, while conceptually simple, would require broad social or institutional consensus to become widely effective.

#### 2.2.2 Information trajectory modeling

This approach modeled how a stream of information should logically evolve over time, allowing deviations from expected patterns to trigger suspicion. For example, weather data from a sensor could be compared to historical norms or predicted forecasts to detect anomalies, much like how financial analysts flag unusual stock price movements.

#### 2.2.3 Ulam games

Inspired by Stanislaw Ulam’s work on adversarial questioning (e.g., “20 Questions” with some deceptive answers), this model explored how to extract truth from a source known to include a limited number of falsehoods. While this approach is more applicable in dialogic or protocol-based contexts such as negotiation or stepwise authentication, it revealed the logic of interacting with a partially compromised information source. Several researchers have investigated this problem, using ideas from error-correcting codes and other areas (Mundici and Trombetta, 1997).

### 2.3 Multiple-source countermeasures

In modern digital ecosystems, most information comes from a mix of sources (e.g., news media, user forums, social networks, etc.). Recognizing this, the Semantic Hacking Project also proposed methods for countering attacks in environments with multiple, potentially colluding actors.

#### 2.3.1 Collaborative filtering and reliability reporting

Borrowed from e-commerce and recommender systems, this method scored information sources based on user feedback and community consensus. While widely used in online commerce to assess vendors, its application in national security settings would involve developing similar trust layers across news sources, intelligence streams, and public discourse.

#### 2.3.2 Byzantine generals models

Adapted from distributed computing, this model examined how to determine the reliability of actors in a group when some participants may be deceitful. Although initially designed for protocol-heavy systems, the model raised important questions about group dynamics in deception detection, especially in coalition-based environments.

#### 2.3.3 Detection of collusion

This approach explored how automated tools could detect coordinated misinformation campaigns. For example, it could analyze message patterns in financial forums to identify multiple posts pushing the same deceptive narrative and flagging suspicious clusters or unusually aligned messaging across supposedly independent accounts.

Interestingly, the team discovered that the challenge wasn’t just detecting outliers, but identifying statistical clusters that mimicked organic diversity but were actually manufactured consensus. Detecting this kind of subtle alignment remains one of the hardest problems in modern disinformation analysis.

#### 2.3.4 Linguistic analysis

This countermeasure used stylometry and other linguistic fingerprinting techniques to determine authorship of supposedly unrelated texts. If dozens of pseudonymous posts share the same stylistic features, there’s a strong chance they were authored by a single source. While less effective for very short content (like tweets), this technique remains powerful when analyzing blogs, articles, or coordinated documents.

### 2.4 Focused case study: the NEI Webworld pump-and-dump operation

Around the time of the Semantic Hacking Project, a particularly vivid example of a semantic (or cognitive) attack was the 1999 NEI Webworld stock manipulation case. A defunct company with little actual value became the center of a disinformation campaign when three individuals bought up shares at $0.05–$0.17 and then used over 500 deceptive messages across Internet message boards to claim the company was on the verge of a lucrative buyout.

By inventing third-party interest and repeating inflated claims through multiple pseudonymous accounts, they drove the share price to $15 within a single day and made $364,000 in profit before selling off their positions.

While technically simple, the attack exploited the perception of independent corroboration and urgency, which was exactly the kind of exploit the Semantic Hacking Project warned about. In this case, several countermeasures proposed by the project might have exposed the deception:

- Collaborative filtering and trust reporting could have lowered the credibility of new or unverified users making bold claims.
- Information trajectory modeling would have flagged the extreme and sudden stock price movement as anomalous.
- Linguistic analysis could have revealed that the hundreds of seemingly independent posts were likely authored by only three people.

The NEI case demonstrated that cognitive attacks are not theoretical. They have financial, legal, and societal impacts. And they are often carried out using nothing more than manipulation of language and identity in open forums (Cybenko et al., 2002c).

### 2.5 Strategic insights for today’s cognitive security landscape

While the Semantic Hacking Project produced technical frameworks and early prototypes, its broader strategic insights offer enduring relevance for today’s AI-powered information terrain. Three themes in particular stand out.

#### 2.5.1 Perception can be weaponized without ever touching the facts

One of the Project’s central realizations was that control over interpretation often matters more than control over content. Cognitive attacks succeed not by hiding or falsifying data, but by subtly steering how people understand it. Many of the proposed countermeasures (e.g., like information trajectory modeling or linguistic clustering) were grounded in the recognition that believable distortions are more dangerous than obvious falsehoods. This principle echoes through today’s influence operations, where AI-generated content increasingly reinforces persuasive frames and synthetic consensus without needing to invent anything outright. The battleground is not information access, but meaning assignment.

#### 2.5.2 Definitions are battlespaces

The Project foresaw the strategic importance of framing and terminology. When key terms like “sentient,” “genocide,” “life,” or “threat” are up for debate, so too are the conclusions that follow from them. Whoever gets to define the terms often shapes the outcome. This battle over semantic framing (which some contemporary DoD staff refer to as “term warfare”) is only becoming more intense in an age where digital repetition, generative AI, and social media can entrench contested definitions rapidly and globally.

#### 2.5.3 Theory itself is a story that can be weaponized

The Project’s deeper insight was that even the frameworks we use to determine what is true (our hypotheses, laws, and scientific narratives) are not immune to manipulation. Theories can be introduced, reframed, or dismissed not only through evidence, but through narrative persuasion. In today’s information landscape, the contest over “truth” is also a contest over what counts as legitimate knowledge. This likely is not just a modern or future of warfare problem; it likely dates back to the earliest forms of warfare and those who knew how to wield information, law, history books, and the “truths” about the heavens were the ones winning the war without firing a shot.

##### 2.5.3.1 From semantic hacking to AI-era cognitive security

Although the Semantic Hacking Project formally concluded in 2003, the intellectual arc it launched has continued to evolve. One major milestone came in 2014, with the AAAI Spring Symposium on “Social Hacking and Cognitive Security on the Internet and New Media,” which convened experts to examine how digital narratives and online behaviors intersected with emerging information threats (AAAI, 2014). Though the term “cognitive security” had not yet fully crystallized, the concepts discussed mirrored many of the Semantic Hacking Project’s insights especially regarding framing, deception, and interpretive manipulation.

Waltzman (2017) delivered congressional testimony that popularized the term “cognitive security” in national security circles (2017). The Information Professionals Association (IPA, 2025) has since grown into a practitioner hub for influence professionals across military, intelligence, academic, and commercial sectors. Meanwhile, NATO began engaging more formally with these ideas through its Innovation Hub and subsequent symposiums. Its 2021 publication, “Countering Cognitive Warfare: Awareness and Resilience,” brought the term into broader use across allied military structures (NATO, 2021).

These developments reflect a growing recognition that the space the Semantic Hacking Project explored around how meaning is manipulated and truth is contested is now the central battleground for information-age conflict.

##### 2.5.3.2 The need for cognitive testbeds

To contextualize how the insights from the Sematic Hacking Project can help with cognitive security issues today, let us go over a few open issues in the field. One of the key practitioner demands emerging today is for cognitive security to develop testbeds and proving grounds similar to those used in cyber and kinetic operations. Just as weapons are stress-tested in controlled environments, influence operations must be simulated, evaluated, and refined outside of live conflict zones (C4ISRNET, 2021).

The Semantic Hacking Project’s suggestion of modeling information trajectories and simulating adversarial questioning (like Ulam Games) could provide a conceptual foundation for these test environments. By building simulations where varying definitions, belief systems, or stimuli are introduced under controlled parameters, researchers could begin developing a formal Test & Evaluation (T&E) doctrine for narrative engagement. This would help institutions gauge not only what messages are persuasive, but “why,” “whom,” and under what shifting contextual conditions.

##### 2.5.3.3 The limits of deepfake detection and the need for psychophysics of belief

Much of today’s discourse on AI and disinformation focuses on detecting deepfakes. But the core issue is not whether something is fake; it’s whether people believe it or not. This gap between reality and perception demands a new line of research: the psychophysics of belief.

The Semantic Hacking Project already emphasized that what counts as “authentic” or “real” can shift based on context, repetition, and source credibility. To build on that, modern cognitive security must investigate which perceptual and semantic qualities make a message seem true (even when it is not) and how those thresholds change across populations and over time. Variables could range from pixel resolution and audio quality (low-level) to perceived authority, body language, or context (high-level). Regular empirical testing across demographic and cultural groups would be required to build models of what people consider trustworthy, how those judgments evolve with exposure, and what kinds of countermeasures might restore healthy skepticism. As the Semantic Hacking Project foreshadowed, even the definition of what is “fake” or “real” is vulnerable to manipulation.

##### 2.5.3.4 Financial and economic warfare in the cognitive age

The NEI Webworld case study foreshadowed a now-mainstream concern: information can move markets. Today’s financial ecosystem, with its heavy reliance on algorithmic trading and AI-driven analysis, is even more susceptible to cognitive manipulation.

A modern systemized example would be the firm Hindenburg Research (2025) that has built a business model around investigating companies and releasing public reports which often triggers massive stock selloffs from perceived fraud or mismanagement. While this form of financial activism may serve a regulatory purpose, its success relies on controlling narrative timing and information asymmetry. On the darker end of the spectrum, scams in the cryptocurrency world (ranging from pump-and-dumps to deepfake-driven wallet theft) demonstrate how deception has become a tactical weapon.

Another notable incident occurred in 2023, when an AI-generated image of an explosion at the Pentagon caused a 15-min dip in the U.S. stock market (New York Times, 2023). That brief but measurable loss revealed how much economic stability now hinges on real-time narrative control. Just as troubling, adversaries may not need to hack infrastructure directly, they can attack trust in the systems that interpret it. The Semantic Hacking Project’s emphasis on collusion detection, trajectory modeling, and information source reliability could provide valuable tools for countering these threats in finance.

## 3 Conclusion: revisiting the past to secure the future

This paper was written not just to highlight an early and underappreciated effort in the study of cognitive security, but to challenge the assumption that today’s problems are wholly new. In the face of fast-moving developments in cognitive security and AI, there is a growing tendency to believe that novel threats require entirely novel solutions. But as the Semantic Hacking Project showed nearly 25 years ago, many of the core dynamics of cognitive conflict were already understood, modeled, and even partially addressed.

We are fortunate to have one of the project’s principal contributors as a co-author, able to preserve and share insights that could easily have been lost. Unfortunately, this kind of historical continuity is rare in defense and national security research. Because so much work is classified, ephemeral, or siloed in closed communities, institutional knowledge often disappears when individuals retire, change roles, or move on. The result is a field where rediscovery often replaces progression, and where the wheel is reinvented more often than we like to admit.

Cognitive security is too important, too urgent for that kind of amnesia. If we are to build effective doctrines, testbeds, and research programs, we must begin by mapping what has already been tried, what was learned, and what still remains unsolved. The Semantic Hacking Project deserves a place in that lineage not as a historical footnote, but as a foundational effort whose questions are still guiding us, even if the technologies around them have changed.

## Data Availability

The original contributions presented in the study are included in the article/supplementary material, further inquiries can be directed to the corresponding author.
